# A statistical framework for analyzing deep mutational scanning data

**DOI:** 10.1186/s13059-017-1272-5

**Published:** 2017-08-07

**Authors:** Alan F. Rubin, Hannah Gelman, Nathan Lucas, Sandra M. Bajjalieh, Anthony T. Papenfuss, Terence P. Speed, Douglas M. Fowler

**Affiliations:** 1 Bioinformatics Division, The Walter and Eliza Hall Institute of Medical Research, Parkville, VIC 3052 Australia; 2 Department of Medical Biology, University of Melbourne, Melbourne, VIC 3010 Australia; 3 Bioinformatics and Cancer Genomics Laboratory, Peter MacCallum Cancer Centre, Melbourne, VIC 3000 Australia; 4 Department of Genome Sciences, University of Washington, Seattle, WA 98195 USA; 5 Institute for Protein Design, University of Washington, Seattle, WA 98195 USA; 6 Department of Pathology, University of Washington, Seattle, WA 98195 USA; 7 Sir Peter MacCallum Department of Oncology, University of Melbourne, Melbourne, VIC 3010 Australia; 8 Department of Mathematics and Statistics, University of Melbourne, Melbourne, VIC 3010 Australia; 9 Department of Bioengineering, University of Washington, Seattle, WA 98195 USA

## Abstract

Deep mutational scanning is a widely used method for multiplex measurement of functional consequences of protein variants. We developed a new deep mutational scanning statistical model that generates error estimates for each measurement, capturing both sampling error and consistency between replicates. We apply our model to one novel and five published datasets comprising 243,732 variants and demonstrate its superiority in removing noisy variants and conducting hypothesis testing. Simulations show our model applies to scans based on cell growth or binding and handles common experimental errors. We implemented our model in Enrich2, software that can empower researchers analyzing deep mutational scanning data.

**Electronic supplementary material:**

The online version of this article (doi:10.1186/s13059-017-1272-5) contains supplementary material, which is available to authorized users.

## Background

Exploring the relationship between sequence and function is fundamental to enhancing our understanding of biology, evolution, and genetically driven disease. Deep mutational scanning is a method that marries deep sequencing to selection among a large library of protein variants, measuring the functional consequences of hundreds of thousands of variants of a protein simultaneously. Deep mutational scanning has greatly enhanced our ability to probe the protein sequence-function relationship [1] and has become widely used [2]. For example, deep mutational scanning has been applied to comprehensive interpretation of variants found in disease-related human genes [3, 4], understanding protein evolution [5–9], and probing protein structure [10, 11] with many additional possibilities on the horizon [2].

In a deep mutational scan, a library of protein variants is first introduced into a model system [12]. Model systems that have been used in deep mutational scanning include phage, bacteria, yeast, and cultured mammalian cells. A selection is applied for protein function or another molecular property of interest, altering the frequency of each variant according to its functional capacity. Selections can be growth-based or implement physical separation of variants into bins, as in phage display or flow sorting of cells. Next, the frequency of each variant in each time point or bin is determined by using deep sequencing to count the number of times each variant appears. Here, the variable region is either directly sequenced using a single-end or paired-end strategy, or a short barcode that uniquely identifies each variant in the population is sequenced instead [12, 13]. Barcoding enables accurate assessment of variable regions longer than a single sequencing read [4, 13, 14]. Analysis of the change in each variant’s frequency throughout the selection yields a score that estimates the variant’s effect. Scoring the performance of individual variants is distinct from a related class of methods that quantify tolerance for change at each position in a target protein [15]. Those approaches enable a different set of biological inferences that we do not seek to address here. Guidelines for the design of deep mutational scanning experiments have been discussed elsewhere [12, 16–18].

Fundamental gaps remain in our ability to use deep mutational scanning data to accurately measure the effect of each variant because practitioners lack a unifying statistical framework within which to interpret their results. Existing methods are diverse in terms of their scoring function, statistical approach, and generalizability. Two established implementations of deep mutational scanning scoring methods, Enrich [19] and EMPIRIC [20], calculate variant scores based on the ratio of variant frequencies before and after selection. This type of ratio-based scoring has been used to quantify the effect of non-coding changes in promoters as well [21]. However, while intuitive and easy to calculate, ratio-based scores are highly sensitive to sampling error when frequencies are low. For experimental designs that sample from more than two time points to improve the resolution of changes in frequency, ratio-based scoring is insufficient so a regression-based approach has been used instead [4, 16, 22, 23]. Both ratio and regression analyses can incorporate corrections for wild-type performance [8, 16, 19, 20, 24] or nonsense variants [20, 22] at the expense of restricting the method to protein-coding targets only.

The lack of a common standard for calculating scores makes comparison between studies difficult and existing bespoke methods are not applicable to the diverse array of experimental designs currently being used. Furthermore, no existing method quantifies the uncertainty surrounding each score, which limits the utility of the data. For example, one of the most compelling applications of deep mutational scanning is to annotate variants found in human genomes with the goal of empowering variant interpretation [4], where estimation of the uncertainty associated with each measurement in a common framework is crucial. At best, current approaches employ ad hoc filtering of putative low-quality scores, often using manually determined read-depth cutoffs.

To address these limitations, we present Enrich2, an extensible and easy-to-use computational tool that implements a comprehensive statistical model for analyzing deep mutational scanning data. Enrich2 includes scoring methods applicable to deep mutational scans with any number of time points. Unlike existing methods, Enrich2 also estimates variant scores and standard errors that reflect both sampling error and consistency between replicates. We explore Enrich2 performance using novel and published deep mutational scanning datasets comprising 243,732 variants in five target proteins, as well as simulated data. We demonstrate that Enrich2’s scoring methods perform better than existing methods across multiple experimental designs. Enrich2 facilitates superior removal of noisy variants and improved detection of variants of small effect and enables statistically rigorous comparisons between variants. Enrich2 is platform-independent and includes a graphical interface designed to be accessible to experimental biologists with minimal bioinformatics experience.

## Results and discussion

### Overview of Enrich2 workflow

**Fig. 1:**
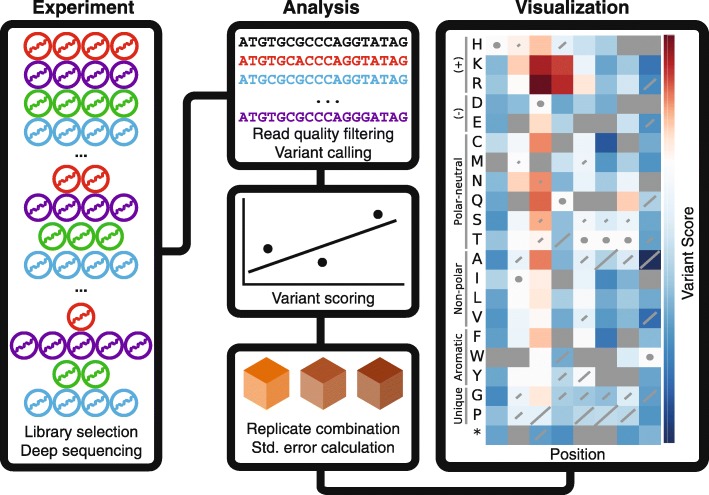
Deep mutational scanning and Enrich2. In a deep mutational scan, a library of protein variants is subjected to selection, which perturbs the frequency of variants. Samples of the library are collected before, during, and after selection and subjected to high-throughput sequencing (*left panel*). Enrich2 processes the high-throughput sequencing files generated from each sample. Sequencing reads are quality filtered and variants are counted by comparing each read to the wild-type sequence. Enrich2 estimates variant scores and standard errors using the variant counts and combines these estimates for replicates (*middle panel*). Enrich2 displays the scores and standard errors as a sequence-function map. A sequence-function map of eight positions of the hYAP65 WW domain is shown (*right panel*). *Cell color* indicates the score for the single amino acid change (*row*) at the given position in the mutagenized region (*column*). Positive scores (in *red*) indicate better than wild-type performance in the assay and negative scores (in *blue*) indicate worse than wild-type performance. *Diagonal lines* in each cell represent the standard error for the score and are scaled such that the highest standard error on the plot covers the entire diagonal. Standard errors that are less than 2% of this maximum value are not plotted. Cells containing *circles* have the wild-type amino acid at that position. *Gray squares* denote amino acid changes that were not measured in the assay

We distilled the common features of a deep mutational scan into a generalized workflow (Fig. 1). After the experiment, each FASTQ file is quality filtered and variants are counted. For directly sequenced libraries, this involves calling the variant for each read (see “Methods”). For barcoded libraries, barcode counts are assigned to variants using an additional file that describes the many-to-one barcode-to-variant relationship. Next, the counts for each variant are normalized and a score is calculated that quantifies the change in frequency of each variant in each selection. Finally, each variant’s scores from replicate selections are combined into a single replicate score using a random-effects model. Variant standard errors are also calculated for each selection and replicate score, allowing the experimenter to remove noisy variants or perform hypothesis testing. Enrich2 is designed to enable users to implement other scoring functions, so long as they produce a score and a standard error. Thus, Enrich2 can serve as a framework for any counting-based enrichment/depletion experiment.

### Scoring a single selection using linear regression

For experimental designs with three or more time points, Enrich2 calculates a score for each variant using weighted linear least squares regression. These time points can be variably spaced, as in samples from a yeast selection withdrawn at different times, or they can be uniformly spaced to represent rounds or bins, as in successive rounds of a phage selection. This method assumes the selection pressure is relatively constant during the course of the selection. Each variant’s score is defined as the slope of the regression line. For each time point in the selection, including the input time point, we calculate a log ratio of the variant’s frequency relative to the wild-type’s frequency in the same time point and regress these values on time. Regression weights are calculated for each variant in each time point based on the Poisson variance of the variant’s count (see “Methods”). We estimate a standard error for each score using the weighted mean square of the residuals about the fitted line. We calculate *p* values for each score using the *z*-distribution under the null hypothesis that the variant behaves like wild-type (i.e. has a slope of 0).

**Fig. 2:**
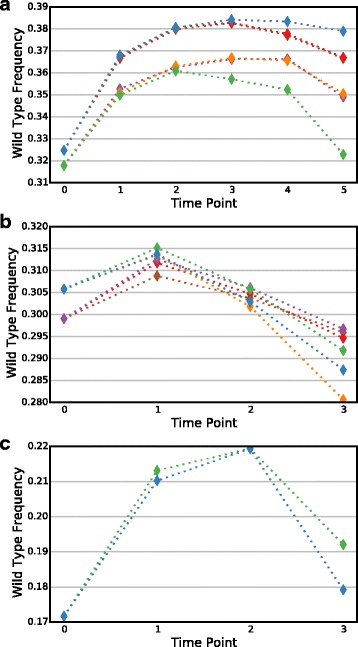
Wild-type frequency can change non-linearly. The change in frequency of the wild-type over the course of replicate selections is shown for (**a**) BRCA1 E3 ubiquitin ligase, (**b**) BRCA1-BARD1 binding, or (**c**) E4B E3 ubiquitin ligase. Each *colored line* represents a single replicate

A problem with linear regression-based scoring is that the wild-type frequency often changes non-linearly over time in an experiment-specific and selection-specific manner (Fig. 2). Some linear model-based approaches subtract the wild-type score from each variant’s score [4, 22], ignoring this issue and potentially reducing score accuracy. A solution for this problem, which has been used extensively, is normalizing each variant’s score to wild-type at each time point [16, 20, 25–27]. We implemented per-time point normalization and compared variant standard errors calculated with and without wild-type normalization for a total of 14 replicates in three different experiments: a phage selection for BRCA1 E3 ubiquitin ligase activity; a yeast two-hybrid selection for BRCA1-BARD1 binding; and a phage selection for E4B E3 ubiquitin ligase activity (Table 1). In all cases, wild-type normalization resulted in significantly smaller variant standard errors (*p* ≈ 0, binomial test, Additional file 1). Variants that remain non-linear after normalization are poorly fit by our regression model and have high standard errors. Thus, they can easily be identified for further examination or removal.

**Table 1:** Datasets analyzed with Enrich2

Target	Assay	Replicates	Time points	Scored variants	Reads (millions)	Run time (h:min)	Reference
BRCA1	Phage display	6	6	11,530	423	6:23	[4]
BRCA1	Yeast two-hybrid	6	4	17,165	306		
E4B	Phage display	2	4	158,939	67	2:27	[14]
Neuraminidase	Growth in cell culture	6^a^	2	6834	24	0:08	[30]
C2 domain	Phage display	3	3	1081	48	1:17	This work
WW domain	Phage display	2^b^	4	48,183	33	10:32	[1]

^a^Three replicate selections each of two experimental conditions, with a shared input library

^b^Resequencing of the same selection

Wild-type normalization is not always the best option. For example, some experimental designs do not have a wild-type sequence in the library, which precludes wild-type normalization. Furthermore, experiments subject to high levels of stochasticity arising from low read depth or limited sampling can benefit from normalization to the total number of reads rather than to wild-type [16]. Normalization to wild-type is also inappropriate in cases where the effect of the wild-type is incorrectly estimated or subject to high levels of error [16, 28]. To deal with these cases, Enrich2 also offers normalization using the number of reads instead of the wild-type count.

**Fig. 3:**
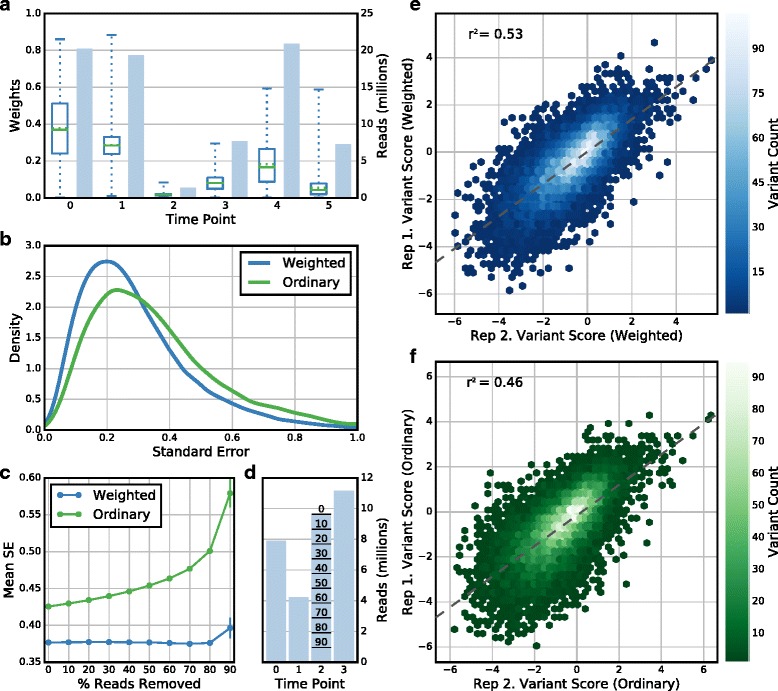
Weighted least squares regression reduces standard error and improves replicate correlation. **a** The number of reads (*shaded blue bars*) and the distribution of variant regression weights (*boxplots*, *solid green line* is the median, *dotted green line* is the mean, *box* spans the first to third quartile, *whiskers* denote the data range) for each time point in a single BRCA1 E3 ubiquitin ligase selection is shown. Time points with fewer reads per variant are downweighted in the regression. The weights for later time points are lower on average because most variants decrease in frequency during the course of the selection. **b** A *density plot* of standard errors for all variants in the selection shown in (**a**) calculated using weighted least squares regression (*blue line*) or ordinary least squares regression (*green line*) is shown. The weighted least squares regression method returns lower standard errors using the same underlying data by minimizing the impact of sampling error in low read count time points. **c** The mean standard error of variants after randomly downsampling reads in a single time point in one of the E4B E3 ubiquitin ligase selections is shown. Mean standard errors for all variants at each read downsampling percentage were calculated using either weighted least squares regression (*blue*) or ordinary least squares regression (*green*). *Error bars* indicate the 95% confidence interval of five random downsampling trials at each percentage. **d** Read counts per time point in the selection described in (**c**) is shown. The *lines* on the *bar* for time point 2 correspond to the level of downsampling on the *x-axis* of (**c**). **e**, **f** *Plots* of variant scores in two replicate selections from the BRCA1 E3 ubiquitin ligase dataset are shown. Replicate agreement for scores calculated using the weighted least squares regression model (**e**) is higher than agreement for scores calculated using ordinary least squares regression (**f**). The *dashed line* shows the line of best fit for the replicate scores in each plot. *Hex color* indicates point density

Wild-type non-linearity is not the only problem in scoring a typical selection. Each time point has a different number of reads per variant and time points with low coverage are more affected by sampling error. An example of this issue is found in one of the replicate selections for BRCA1 E3 ubiquitin ligase activity (Fig. 3a). To address this problem, Enrich2 downweights time points in the regression with low counts per variant. Without weighted regression, the experimenter is forced to choose between three undesirable options: using the low coverage time point and adding noise to the measurements; removing the time point and complicating efforts to compare replicates; or spending time and resources to re-sequence the time point. Weighting avoids these undesirable options, achieving lower variant standard errors as compared to ordinary regression (Fig. 3b). To show that this effect is general and not a feature of the specific BRCA1 replicate we analyzed, we downsampled reads from a single time point in the E4B E3 ubiquitin ligase dataset. We find that weighted regression reduces the mean standard error regardless of the fraction of reads removed (Fig. 3c, d). Finally, we show that weighted regression improves reproducibility between replicates in the BRCA1 E3 ubiquitin ligase dataset, even in the absence of any filtering (Fig. 3e, f). A previously developed Bayesian MCMC approach could be used to generate a posterior variance, which would be of similar value to our standard errors [28]. However, this approach would be impracticably slow for tens of thousands of variants.

For experiments with only two sequenced populations or time points (e.g. “input” and “selected”), Enrich2 calculates the slope between the two time point log ratios, which is equivalent to frequently used ratio-based scoring methods [1, 19, 20, 24]. Unlike previous implementations of ratio-based scoring, we provide standard error estimates for each score using Poisson assumptions (see “Methods”).

### A random-effects model for scoring replicate selections

**Fig. 4:**
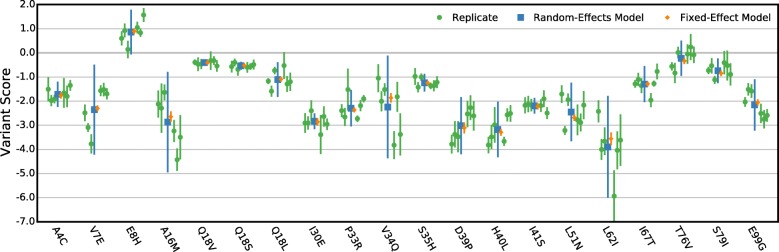
A random-effects model for scoring replicate selections. Variant scores for 20 randomly selected variants from the BRCA1 E3 ubiqutin ligase dataset are shown. The replicate scores (*green*) were determined for each variant using Enrich2 weighted regression. Combined variant score estimates were determined using a fixed-effect model (*orange*) or the Enrich2 random-effects model (*blue*). In all cases, *error bars* show +2 or –2 standard errors

Deep mutational scans are affected by various sources of error in addition to sampling error. One way to deal with this problem is to perform replicates. Usually, each variant’s score is calculated by taking the mean across replicates, which ignores the distribution of replicate scores. Furthermore, if an error is calculated, it is derived only from the replicate scores’ distribution and ignores any error associated with each replicate score. One alternative is to combine replicate scores using a fixed-effect model [29]. We examined this approach for the BRCA1 E3 ubiquitin ligase dataset (Fig. 4) and found that because variant scores can vary widely between replicates, this method dramatically underestimates the standard error of the combined variant score. We therefore implemented a random-effects model that estimates each variant’s score based on the distribution of that variant’s scores across all replicates. This random-effects model also produces a standard error estimate for each variant that captures selection-specific error as well as error arising from the distribution of replicate scores (see “Methods”).

The random-effects model furnishes variant scores that are less sensitive to outlier replicates than a fixed-effect model (Fig. 4). Additionally, standard errors estimated by the random-effects model better reflect the distribution of replicate scores, providing a better basis for subsequent hypothesis testing. The same random-effects model can be used for experiments with any number of time points or replicates or with any Enrich2 scoring function (Additional file 2: Figure S1). A key advantage of this approach is that error is quantified on a per-variant basis, unlike the usual approach of comparing replicate selections using pairwise correlation [4, 15, 22]. This allows experimenters to use replicate data to make inferences about individual variants, rather than simply as a quality control check for whole experiments.

### Standard error-based variant filtering

Per-variant standard error estimates enable the removal of variants with unreliable scores. This contrasts with previous filtering schemes, which employed an empirical cutoff for the minimum number of read counts for each variant in the input library or throughout the selection [1, 4, 12, 14, 30–36]. Read count cutoffs eliminate low-count variants that may be unreliably scored due to sampling error, but ignore other sources of noise and may introduce a bias against variants that become depleted after selection. Enrich2 retains low-count variants and enables the experimenter to determine which scores are reliable directly from the associated standard error.

**Fig. 5:**
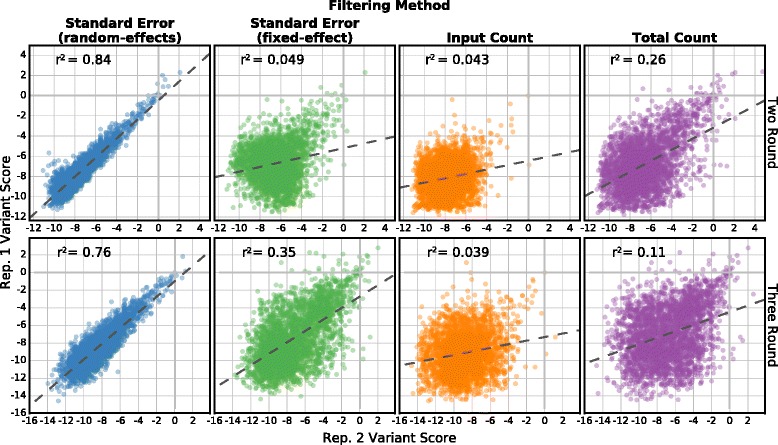
Standard error-based filtering improves replicate correlation. Variant scores from two replicates of the C2 domain dataset are shown. Each *panel* plots the top quartile of variants selected by standard error from the random-effects model (*leftmost column*, *blue points*), standard error from the fixed-effect model (*middle-left column*, *green points*), input library count (*middle-right column*, *orange points*), or total count in all libraries (*rightmost column*, *purple points*). Scores and standard errors are calculated using only the input and final round of selection (*top row*) or using all three rounds (*bottom row*). The *dashed line* is the best linear fit and the Pearson correlation coefficient is shown

To assess whether standard error-based filtering performs better than read count-based filtering, we analyzed data from a deep mutational scan of the C2 domain of Phospholipase A2 (Table 1). Here, a library of 84,252 phage-displayed C2 domain variants was selected for lipid binding over several rounds. This dataset was un-analyzable using previous methods due to the apparent extreme variability between replicate selections. We compared filtering based on four different parameters: variant standard error calculated using the random-effects model or the fixed-effect model, read count in the input round, and total read count in all rounds of selection. To quantify filtering method performance, we took the top quartile of variants selected by each filtering method. Then, we calculated the pairwise Pearson correlation coefficient between variant scores for each possible pair of the three replicates in the C2 domain dataset (Fig. 5, Additional file 3). We found that filtering based on standard errors from the random-effects model was the only method that recovered a replicable subset of variants from this dataset. In fact, input count filtering selected a subset of variants whose scores were more poorly correlated than the unfiltered set. We performed a similar analysis on the higher-quality E4B, neuraminidase, and BRCA1 replicate datasets using the top three quartiles of variants. As for the C2 domain, we found that filtering based on random-effects standard error outperforms the other filtering methods (Additional file 3). For example, in the E4B dataset random-effects standard error filtering performed better (pairwise Pearson r^2^ = 0.80) than fixed-effect standard error (r^2^ = 0.59), input library count (r^2^ = 0.58), or total count filtering (r^2^ = 0.59). We note that any filtering strategy removes variants and reduces coverage. To explore how the stringency of variant filtering affects replicate correlation, we calculated replicate correlations after removing increasing numbers of variants according to each of the four filtering methods (Additional file 2: Figure S2). We found that filtering by standard errors from the random-effects model was the only approach that yielded high correlations between replicates for the C2 domain data. Furthermore, random-effects standard error filtering performed better at nearly all filtering stringencies in both the C2 domain and BRCA1 E3 datasets.

**Fig. 6:**
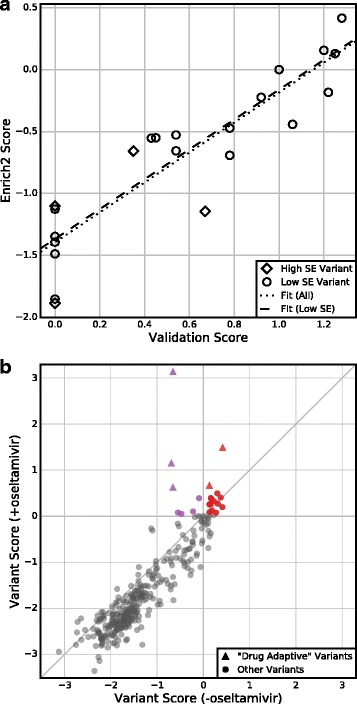
Standard errors enable hypothesis testing. **a** Enrich2 variant scores are plotted against single-variant growth assay scores for the 22 individually validated variants of the neuraminidase dataset. Four (18%) of these variants have Enrich2 standard errors larger than the median standard error. The *dotted line* shows the best linear fit for all variants and the *dashed line* shows the best linear fit for variants with standard errors less than the median. **b** Enrich2 variant scores are plotted for selections performed in the presence or absence of the small molecule inhibitor oseltamivir. *Colored points* indicate variants that significantly outperformed wild-type in the drug’s presence. *Red points* also scored significantly higher than wild-type in the drug’s absence. *Triangles* indicate the five “drug-adaptive” mutations identified originally [30]

To further demonstrate the utility of Enrich2 standard error-based filtering, we re-analyzed a deep mutational scan of the influenza virus neuraminidase gene (Table 1). In this experiment, 22 neuraminidase variants were individually validated and used to assess the quality of the deep mutational scanning data. Of these individually validated variants, four had large variant score standard errors as determined by Enrich2’s random-effects model (Fig. 6a, Additional file 2: Figure S3, Additional file 4). Removing these high-standard error variants improved the correlation between the deep mutational scanning scores and individual validation scores from Pearson r^2^ = 0.81 to r^2^ = 0.87. Removal of these variants also improved the correlation when scores were calculated as originally described in the study (Pearson r^2^ = 0.80 versus r^2^ = 0.84) (Additional file 2: Figure S3) [30]. This suggests that scores of variants with low Enrich2 standard errors are more likely to reflect the results of gold standard validation experiments and supports the use of standard error-based filtering for selecting candidate variants for follow-up studies. We note that in the neuraminidase experiment, the three replicates used a common starting library. This design fails to capture some artifacts, especially those introduced during cloning. Ideally, full biological replicates should be collected.

### Standard error-based hypothesis testing

An important challenge in analyzing deep mutational scanning data is determining whether a variant behaves differently from wild-type or differently under altered conditions. Enrich2 standard errors empower experimenters to perform statistical tests for such differences. By default, Enrich2 calculates raw *p* values for each score under the null hypothesis that the variant’s score is indistinguishable from wild-type using a *z*-test. This allows the user to discriminate between variants with extreme scores due to sampling error or other noise from those that are confidently estimated to be different from wild-type. We note that Enrich2 provides raw *p* values and users should correct for multiple testing using their preferred method.

We can also use a *z*-test to determine whether variants have different functional consequences under altered experimental conditions. For example, deep mutational scans of the neuraminidase gene were conducted in the presence and absence of the small molecule neuraminidase inhibitor oseltamivir (Table 1). The original study identified five “drug-adaptive” variants, defined as those that outperformed wild-type in the presence of oseltamivir [30]. These five drug-adaptive variants included three known oseltamivir-resistant variants. In our reanalysis, we identified 22 drug-adaptive variants including all five variants found in the original study (Fig. 6b, Additional file 5). Fifteen of these 22 drug-adaptive variants also had a significantly higher score than wild-type in the absence of the inhibitor and therefore might be more likely to occur in natural virus populations. Our results agree broadly with the original analysis. By using Enrich2 to calculate scores and standard errors for variants across replicates, we were able to identify additional candidate variants with small but statistically significant effects, some of which could be of biological interest. Of course, any new candidate variants could be false positives and they would need to be individually validated, as was done in the original study.

### Simulations of deep mutational scanning data

Our analyses of experimental data suggest that Enrich2 is a useful tool for exploring and understanding deep mutational scanning datasets. In support of this, we generated simulated datasets with predetermined variant effects and compared mathematically predicted Enrich2 variant effect scores to scores calculated from simulated data. Using this approach, we demonstrate that the Enrich2 method can be applied to data from either cell growth or binding assays and can handle different types of noise.

Deep mutational scanning datasets can be generated using different selection assays. Nearly all scans employ either cell growth assays or binding assays, which are typically conducted using phage or yeast display [12]. To demonstrate that the Enrich2 method can meaningfully assign variant scores for both assay types, we simulated data where each variant’s true effect was predetermined (see “Methods”). In growth simulations, a variant’s true effect was the growth rate of a cell carrying that variant; in binding simulations, a variant’s true effect was the probability of a cell or phage carrying that variant progressing to the subsequent round of selection. In our simulations, each variant’s true effect was drawn from a normal distribution with the wild-type true effect in the 75th percentile of the distribution, which is consistent with empirical datasets (Additional file 6).

**Fig. 7:**
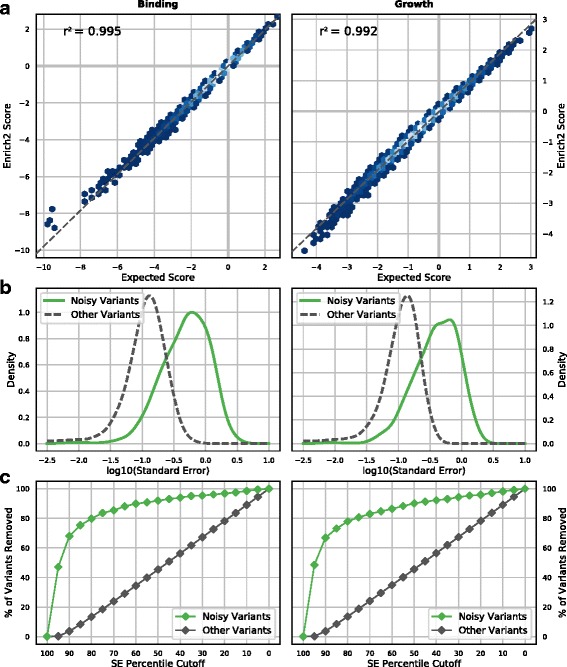
Variant scoring for growth and binding experiments using simulated data. **a** Enrich2 variant effect scores derived from simulated data are plotted against expected Enrich2 scores based on true variant effects in the simulation. Enrich2 accurately scores variants in both simulated binding assays (*left*) and growth assays (*right*). *Shading* indicates point density from low (*blue*) to high (*white*). **b** Noisy variants were generated by randomizing their true effect in one replicate selection (*green line*). Noisy variants have higher overall standard errors than other variants (*dashed gray line*) in both binding and growth assay simulations. **c** The percentage of variants removed at each standard error percentile cutoff (5% intervals) is plotted. Standard error filtering preferentially removes noisy variants (*green points*)

Each simulated dataset contained 10,000 unique variants including wild-type. For each selection, a starting variant population was independently generated and then five rounds of growth or binding selection were performed (see “Methods”). Five replicate selections were simulated for each dataset. Sequencing was simulated such that each variant had, on average, 200 reads. The resulting datasets were scored by Enrich2 using the weighted least squares regression method and replicates were combined using the random-effects model. We found that the Enrich2 scores are strongly correlated with predicted scores based on the true variant effects (r^2^ = 0.995 for binding and r^2^ = 0.992 for growth) (Fig. 7a). Thus, the Enrich2 method captures true variant effects for both growth-based and binding-based assays. We note that the relationship of these variant effects to a physical parameter of interest (e.g. *K*_*d*_ for binding) depends on the specific conditions of the experiment [37–39].

We also simulated noisy data and evaluated Enrich2’s ability to identify affected variants. One type of noise is inconsistent variant effects between replicates, which can arise from cloning errors or experimental variation. We simulated datasets in which 2% of variants in each of the five biological replicates were randomly assigned a new true effect. As expected, noisy variants have higher standard errors than other variants (Fig. 7b) and standard error-based filtering is an effective tool for removing them (Fig. 7c). The magnitude of a noisy variant’s standard error is proportional to the magnitude of the difference between the variant’s original true effect and the resampled true effect (r^2^ = 0.85 for binding and r^2^ = 0.93 for growth; Additional file 2: Figure S4). Another type of noise arises from unexpected amplification or depletion of variant counts in a single time point, which can be due to polymerase chain reaction (PCR) jackpotting or other artifacts during the DNA isolation, amplification, and sequencing steps. We simulated datasets in which 10% of variants are over-represented or under-represented in a single time point. We found that the random-effects model accurately assigns scores to these amplified or depleted variants (Additional file 2: Figure S5A) and the affected variants are easily identified by their replicate standard errors (Additional file 2: Figure S5B). These results illustrate that the Enrich2 method is robust to common types of noise present in deep mutational scanning data.

## Conclusions

We developed a statistical framework for analyzing deep mutational scanning data that is applicable to many common experimental designs. We showed that our statistical method is superior to existing methods for removing noisy variants and detecting variants of small effect, enabling researchers to extract more from their datasets. We implemented our method in Enrich2, a computationally efficient graphical software package intended to improve access to deep mutational scanning for labs without data analysis experience. Enrich2 is extensible, so users can implement and easily share new scoring functions as new deep mutational scanning experimental designs are developed.

Enrich2 builds upon previous approaches to regression-based scoring, which we improved in two ways. First, per-time-point wild-type normalization helps reduce the effects of non-linear behavior under the assumption that many sources of non-linearity affect most variants similarly. Second, weighting each regression time point based on variant counts helps alleviate sampling error. In addition to these improvements, Enrich2 combines replicate selections into a single set of variant scores with standard errors to help identify variants that behave consistently in a given assay. Though variant score precision does not guarantee accuracy, we showed that removing variants with high standard errors from the neuraminidase dataset did improve the correlation between deep mutational scanning results and gold-standard measurements.

Enrich2 furnishes generalized variant effect scores, which we showed are applicable to both growth-based and binding-based deep mutational scans. In the case of growth-based deep mutational scans, variant scores are linearly related to growth rate. In the case of binding-based deep mutational scans, variant scores are linearly related to the log of the likelihood of selection in each round. We note that the relationship between the likelihood of selection and variant binding affinity depends on experimental specifics including the number of molecules displayed per cell or phage, ligand concentration, and degree of non-specific binding [39]. Furthermore, the regression-based approach described here is designed for deep mutational scans with constant selection pressure. Selections conducted over longer timescales or selections in which the selection pressure is modulated by the experimenter may not be modeled accurately by our approach [8, 40, 41]. Specific scoring methods that take into account experimental details such as ligand concentration or variable selection pressure could easily be added to Enrich2, taking advantage of the program’s existing read counting, variant calling, replicate combining, and visualization machinery.

Enrich2 standard errors can also be used to conduct hypothesis tests comparing variants within a single experimental condition or between multiple conditions. When comparing variants between conditions, we assume that the distribution of scores between conditions is roughly similar, but this assumption does not hold in all cases. For example, the shape of the score distribution is a function of the strength of the selective pressure applied [8] and, more generally, the experimental conditions employed. Thus, Enrich2 standard errors should be used with caution when comparing variants between differing selections unless the variant scores are similarly distributed and the selection conditions are comparable. A general method for normalizing scores to facilitate comparisons across different conditions or selection pressures remains an important open question, as existing approaches are computationally intensive [28].

The use of deep mutational scanning is expanding rapidly and better tools for analysis will help it flourish. As with other widely used high-throughput experimental methods, a robustly implemented common statistical framework reduces barriers to entry, ensures data quality, and enables comparative analyses. We suggest that Enrich2 can help deep mutational scanning continue to grow by providing a foundation for meeting these challenges and facilitating further exploration and collaboration.

## Methods

### Variant calling and sequence read handling

Enrich2 implements alignment-free variant calling. Variant sequences are expected to have the same length and start point as the user-supplied wild-type sequence, which allows Enrich2 to compare each variant to the wild-type sequence in a computationally efficient manner. In addition to this alignment-free mode, an implementation of the Needleman-Wunsch global alignment algorithm [42] is included that will call insertion and deletion events. Enrich2 supports overlapping paired-end reads and single-end reads for direct variant sequencing, as well as barcode sequencing for barcode-tagged variants.

### Calculating enrichment scores

For selections with at least three time points, we define *T*, which includes all time points, and *T*′, which includes all time points except the input (*t*_0_). The frequency of a variant (or barcode) *v* in time point *t* is the count of the variant in the time point (*c*_*v*,*t*_) divided by the number of reads sequenced in the time point (*N*_*t*_).

$$ {f}_{v,t}=\frac{c_{v,t}}{N_t} $$

The change in frequency for a variant *v* in a non-input time point *t* ∊ *T*′ is the ratio of frequencies for *t* and the input.

$$ {r}_{v,t}=\frac{f_{v,t}}{f_{v,0}} $$

Instead of using this raw change in variant frequency, we divide each variant’s ratio by the wild-type (*wt*) variant’s ratio.

$$ \frac{r_{v,t}}{r_{wt,t}}=\frac{c_{v,t}{c}_{wt,0}}{c_{v,0}{c}_{wt,t}} $$

Because the library size terms (*N*_*t*_ and *N*_0_) in the frequencies cancel out, the ratio of ratios is not dependent on other non-wild-type variants in the selection. In practice, we add $\frac{1}{2}$ to each count to assist with very small counts [43] and take the natural log of this ratio of ratios.

$$ {L}_{v,t}= \log \left(\frac{\left({c}_{v,t}+\frac{1}{2}\right)\left({c}_{wt,0}+\frac{1}{2}\right)}{\left({c}_{v,0}+\frac{1}{2}\right)\left({c}_{wt,t}+\frac{1}{2}\right)}\right) $$

This equation can be rewritten as

$$ {L}_{v,t}= \log \left(\frac{c_{v,t}+\frac{1}{2}}{c_{wt,t}+\frac{1}{2}}\right)- \log \left(\frac{c_{v,0}+\frac{1}{2}}{c_{wt,0}+\frac{1}{2}}\right) $$

If we were to regress *L*_*v*,*t*_ on *t* ∊ *T* ^′^, we note that the second term is shared between all the time points and therefore only affects the intercept of the regression line. We do not use the intercept in the score, so instead we regress on *M*_*v*,*t*_ and use all values of *t* ∊ *T*.

$$ {M}_{v,t}= \log \left(\frac{c_{v,t}+\frac{1}{2}}{c_{wt,t}+\frac{1}{2}}\right) $$

The score is defined as the slope of the regression line, ${\widehat{\beta}}_v$. In practice, we regress on $\frac{t}{ \max T}$ to facilitate comparisons between selections with different magnitudes of time points (e.g. 0/1/2/3 rounds versus 0/24/48/72 hours).

To account for unequal information content across time points with variable sequencing coverage, we perform weighted linear least squares regression [44]. The regression weight for *M*_*v*,*t*_ is *V*_*v*,*t*_^−1^, where *V*_*v*,*t*_ is the variance of *M*_*v*,*t*_ based on Poisson assumptions [43] and is approximately

$$ {V}_{v,t}=\frac{1}{c_{v,t}+\frac{1}{2}}+\frac{1}{c_{wt,t}+\frac{1}{2}} $$

For selections with only two time points (e.g. input and selected), we use the slope of the line connecting the two points as the score. This is equivalent to the wild-type adjusted log ratio (*L*_*v*_) derived similarly to *L*_*v*,*t*_ above.

$$ {L}_v= \log \left(\frac{c_{v,sel}+\frac{1}{2}}{c_{wt,sel}+\frac{1}{2}}\right)- \log \left(\frac{c_{v,inp}+\frac{1}{2}}{c_{wt,inp}+\frac{1}{2}}\right) $$

As there is no residual error about the fitted line, we must use a different method to estimate the standard error. We calculate a standard error (*SE*_*v*_) for the enrichment score *L*_*v*_ under Poisson assumptions [24, 43].

$$ S{E}_v=\sqrt{\frac{1}{c_{\mathit{v,inp}}+\frac{1}{2}}+\frac{1}{c_{\mathit{wt,inp}}+\frac{1}{2}}+\frac{1}{c_{\mathit{v,sel}}+\frac{1}{2}}+\frac{1}{c_{\mathit{wt,sel}}+\frac{1}{2}}} $$

For experiments with no wild-type sequence, scores can be calculated using the filtered library size for each time point *t*, which is defined as the sum of counts at time *t* for variants that are present in all time points.

### Combining replicate scores

To account for replicate heterogeneity, we use a simple meta-analysis model with a single random effect to combine scores from each of the *n* replicate selections into a single score for each variant. Each variant’s score is calculated independently. Enrich2 computes the restricted maximum likelihood estimates for the variant score ($\widehat{\beta}$) and standard error (${\widehat{\sigma}}_s$) using Fisher scoring iterations [45]. Given the replicate scores (${\widehat{\beta}}_i$) and estimated standard errors (${\widehat{\sigma}}_i$) where *i* = 1, 2, …, *n*, the estimate for $\widehat{\beta}$ at each iteration is the weighted average:

$$ \widehat{\beta}=\frac{{\displaystyle {\sum}_{i=1}^n}{\widehat{\beta}}_i{\left({\widehat{\sigma}}_s^2+{\widehat{\sigma}}_i^2\right)}^{-1}}{{\displaystyle {\sum}_{i=1}^n}{\left({\widehat{\sigma}}_s^2+{\widehat{\sigma}}_i^2\right)}^{-1}} $$

The starting value for ${\widehat{\sigma}}_s^2$ at the first iteration is:

$$ {\widehat{\sigma}}_s^2=\frac{1}{n-1}{\displaystyle \sum_{i=1}^n}{\left({\widehat{\beta}}_i-\overline{\widehat{\beta}}\right)}^2 $$

Enrich2 calculates the following fixed-point solution for ${\widehat{\sigma}}_{s+1}^2$:

$$ {\widehat{\sigma}}_{s+1}^2={\widehat{\sigma}}_s^2\frac{{\displaystyle {\sum}_{i=1}^n}{\left({\widehat{\sigma}}_s^2+{\widehat{\sigma}}_i^2\right)}^{-2}{\left({\widehat{\beta}}_i-\widehat{\beta}\right)}^2}{{\displaystyle {\sum}_{i=1}^n}{\left({\widehat{\sigma}}_s^2+{\widehat{\sigma}}_i^2\right)}^{-1}-\frac{{\displaystyle {\sum}_{i=1}^n}{\left({\widehat{\sigma}}_s^2+{\widehat{\sigma}}_i^2\right)}^{-2}}{{\displaystyle {\sum}_{i=1}^n}{\left({\widehat{\sigma}}_s^2+{\widehat{\sigma}}_i^2\right)}^{-1}}} $$

Because it is more computationally efficient to perform a fixed number of iterations for all variant scores in parallel than to test for convergence of each variant, Enrich2 performs 50 Fisher scoring iterations. In practice, this is more than sufficient for ${\widehat{\sigma}}_s^2$ to converge. We record the difference ${\varepsilon}_s={\widehat{\sigma}}_s^2-{\widehat{\sigma}}_{s-1}^2$ for the final iteration and identify any variants with high values for *ɛ*_*s*_ as variants that failed to converge. No such variants were encountered in the analyses detailed here.

For the fixed-effect model [29], we calculate the variant score (${\widehat{\beta}}^{\mathit{\hbox{'}}}$) and standard error (${\widehat{\sigma}}_s^{\mathit{\hbox{'}}}$) using a weighted average of the replicate scores (${\widehat{\beta}}_i$) where the weight for each score is the inverse of that variant’s variance (${\widehat{\sigma}}_{s-1}^2$). The standard error of the variant ${\widehat{\sigma}}_s^{\mathit{\hbox{'}}}$ is:

$$ {\widehat{\sigma}}_s^{\hbox{'}}=\sqrt{\frac{1}{{\displaystyle {\sum}_{i=1}^n}{\widehat{\sigma}}_i^{-2}}} $$

The fixed-effect model was used for comparison purposes only and is not implemented in the Enrich2 software.

### Derivation of predicted scores

The behavior of a variant *v* in a simulated binding experiment (e.g. phage display, yeast display) can be described in terms of the displaying entity’s likelihood of being selected in a given round [39, 46]. This likelihood is related to the binding affinity of each variant, and, by extension, the binding probability of an individual protein molecule under the experimental conditions. The relationship between variant binding affinity, monomer binding probability, and likelihood of selection will depend on the specifics of the experiment such as the number of molecules displayed per cell or phage, ligand concentration, and non-specific binding [39]. Each round of selection is a time point *t* in the analysis, so we can assign each variant a probability of being selected in a given time point (*p*_*v*,*t*_). We assume that *p*_*v*,*t*_ = *p*_*v*,0_ = *p*_*v*_ (i.e. that the probability is constant throughout the selection) and that any grow out or amplification steps are uniform across all variants.

The initial variant population is determined by the variant population frequencies (${f}_{v,0}^{\mathit{\prime}}$) and the size of the starting population (${N}_0^{\prime }$).

$$ {c}_{v,\ 0}^{\prime }={f}_{v,\ 0}^{\prime }{N}_0^{\prime } $$

We note that ${c}_{v,\mathrm{t}}^{\mathit{\prime}}$, ${f}_{v,\mathrm{t}}^{\mathit{\prime}}$, and ${N}_{\mathrm{t}}^{\prime }$ refer to the variant population itself, in contrast to the previously defined *c*_*v*,*t*_, *f*_*v*,*t*_, and *N*_*t*_, which refer to sequence reads derived from the variant population.

We define *a*_*t*_ as a factor describing growth between round *t* and the previous round (*a*_0_ = 1). We assume that *a*_*t*_ is the same for all variants. The count for a variant in time point *t*+1 in terms the count in time point *t* is:

$$ {c}_{v,\ t+1}^{\prime }={a}_{t+1}{p}_v{c}_{v,\ t}^{\prime } $$

Therefore, the count for a variant in time point *t* given the starting count is:

$$ {c}_{v,t}^{\hbox{'}}={c}_{v,0}^{\hbox{'}}{\displaystyle \prod_{j=1}^t}{a}_j{p}_v={f}_{v,0}^{\hbox{'}}{N}_0^{\hbox{'}}{p}_v^t{\displaystyle \prod_{j=1}^t}{a}_j $$

We can write the ratio of variant counts in these terms and define the log ratio for binding experiments (*M*_*v*,*t*_^'^).

$$ \frac{c_{v,t}^{\hbox{'}}}{c_{wt,t}^{\hbox{'}}}=\frac{f_{v,0}^{\hbox{'}}{N}_0^{\hbox{'}}\kern0.1em {p}_v^t{\displaystyle {\prod}_{j=1}^t}{a}_j}{f_{wt,0}^{\hbox{'}}{N}_0^{\hbox{'}}\kern0.5em {p}_{wt}^t{\displaystyle {\prod}_{j=1}^t}{a}_j}=\frac{f_{v,0\kern0.1em }^{\hbox{'}}{p}_v^t}{f_{wt,0\kern0.1em }^{\hbox{'}}{p}_{wt}^t} $$

$$ {M}_{v,t}^{\hbox{'}}= \log \left(\frac{c_{v,t}^{\hbox{'}}}{c_{wt,t}^{\hbox{'}}}\right)=t\cdot \log \left(\frac{p_v}{p_{wt}}\right)+ \log \left(\frac{f_{v,0}^{\hbox{'}}}{f_{wt,0}^{\hbox{'}}}\right) $$

If we substitute *t* for ${t}^{\prime }=\frac{t}{ \max \kern0.3em T}$, we find that the expected score for binding experiments under the regression scoring model (${\beta}_v^{\prime }$) should be related to the variant selection probability (*p*_*v*_) by:

$$ {\beta}_v^{\prime }=\left( \max \kern0.3em T\right) \log \left(\frac{p_v}{p_{wt}}\right) $$

The behavior of a variant *v* in a simulated growth experiment can be described by the growth rate at time *t* (*μ*_*v*_(*t*)). Unlike in the round-based binding experiment case, time in growth experiments is modeled as continuous. We assume that *μ*_*v*_(*t*) = *μ*_*v*_(0) = *μ*_*v*_ (i.e. that the growth rate is constant throughout the selection) and that any amplification steps are uniform across all variants. This derivation is based on [16, 18]. In interference-free growth, the growth of individual variants can be described by the first order equation:

$$ \frac{d{c}_v^{\hbox{'}}}{dt}={\mu}_v{c}_v^{\hbox{'}}(t) $$

Therefore, the count for a variant at time *t* given the starting count is:

$$ {c}_v^{\hbox{'}}(t)={c}_{v,0}^{\hbox{'}}{e}^{\mu_vt}={f}_{v,0}^{\hbox{'}}{N}_0^{\hbox{'}}{e}^{\mu_vt} $$

We can write the ratio of variant counts in these terms and construct the continuous function *M*_*v*_^″^(*t*).

$$ \frac{c_v^{\hbox{'}}(t)}{c_{wt}^{\hbox{'}}(t)} = \frac{N_0^{\hbox{'}}\kern0.2em {f}_{v,0}^{\hbox{'}}{e}^{\mu_vt}}{N_0^{\hbox{'}}\kern0.2em {f}_{wt,0}^{\hbox{'}}{e}^{\mu_{wt}t}} = \frac{f_{v,0}^{\hbox{'}}}{f_{wt,0}^{\hbox{'}}}{e}^{\left({\mu}_v-{\mu}_{wt}\right)t} $$

$$ {M}_v^{{\prime\prime} }(t)= \log \left(\frac{c_v^{\hbox{'}}(t)}{c_{wt}^{\hbox{'}}(t)}\right)=\left({\mu}_v-{\mu}_{wt}\right)t+ \log \left(\frac{f_{v,0}^{\hbox{'}}}{f_{wt,0}^{\hbox{'}}}\right) $$

We convert to the discrete function *M*_*v*,*t*_^″^ for convenience by assuming that *m* timepoints are sampled at constant intervals, determined by the number of wild-type doublings (*δ*) per time point, such that max *T* = *mδ*. We then find that the expected score for growth experiments under the regression scoring model (*β*_*v*_^″^) should be related to the growth rate (*μ*_*v*_) by:

$$ {\beta}_v^{{\prime\prime} }=m\delta \left({\mu}_v-{\mu}_{wt}\right) $$

### Generation of simulated datasets

Simulated datasets contain 10,000 unique variants (including wild-type), each characterized by a true variant effect: the probability of selection in each round (*p*_*v*_) for binding simulations or the growth rate (*μ*_*v*_) for growth simulations. We assume that the variant effect distribution is normal and set the wild-type effect to *p*_*wt*_ = 0.5 and *μ*_*wt*_ = 1. We set the wild-type effect at the 75th percentile of the distribution and set the standard deviation to 0.1. We draw 9999 variants from this distribution, with 0.05 < *p*_*v*_ < 0.99 and 0.05 < *μ*_*v*_ < 5.

In each case, the population size is 10 million, with a starting wild-type frequency of 1%. Starting counts for each variant are simulated using a log-normal distribution of variant counts in the input time point such that the mean variant input count is 990 and the standard deviation of the distribution is 0.4 [16, 47]. Starting counts are independently generated for each replicate.

For each replicate, the starting population undergoes five rounds of selection. The count of each variant after binding (*k*_*v*,*t*_) is generated using a binomial distribution with parameters *n* = *c*_*v*,*t*−1_^'^ and *p* = *p*_*v*_. The count of each variant after growth (*g*_*v*,*t*_) is generated using a negative binomial distribution with parameters *r* = *c*_*v*,*t*−1_^'^ and $p={e}^{-{\mu}_v\Delta t}$, $\Delta t=\frac{\delta \ln 2}{\mu_{wt}}$. For these simulations, *δ* = 2. The population count for each variant (*c*_*v*,*t*_^'^) is obtained by performing weighted random sampling with replacement, where the weight for each variant is proportional to *k*_*v*,*t*_ or *g*_*v*,*t*_ and the total population size was 10 million.

Read counts for each variant (*c*_*v*,*t*_) are simulated by performing weighted random sampling with replacement, where the weight for each variant is proportional to the population counts (*c*_*v*,*t*_^'^) and the average sequencing depth is 200 reads per variant (approximately 2 million reads per time point).

We simulate replicate noise by drawing a new variant effect from the variant effect distribution for 10% of variants (not including wild-type). These noisy variants were randomly chosen. This new variant effect was used to simulate one replicate and the other four replicates used the original effect. Noisy effects were split uniformly between the five replicates, such that 2% of the variants in each replicate were affected.

We simulate time point amplification and depletion noise by multiplying or dividing *c*_*v*,*t*_^'^ by 50 before performing the sampling step to obtain *c*_*v*,*t*_. We randomly choose 10% of variants to be affected by noise, 5% subject to amplification and 5% subject to depletion, split uniformly among the five replicates. For each noisy variant in the chosen replicate, one time point (including input) was randomly chosen for amplification or depletion.

Python code for generating these simulated datasets is available as simdms v0.1 (DOI: 10.5281/zenodo.546311).

### Deep mutational scan of Phospholipase A2

A region proximal to both lipid binding sites of the C2 domain of Phospholipase A2 (PLA2) was targeted for deep mutational scanning. Positions 94–97 of the C2 domain of mouse PLA2-alpha (ANYV) were fully randomized using a doped synthetic oligonucleotide. The library of C2 subdomains containing mutations was cloned into the AvrII and PpuMI sites of wild-type C2 domain in pGEM. The library was subcloned into phage arms and expressed on the surface of bacteriophage using the T7 phage display system according to the manufacturer’s instructions (Novagen T7Select 10-3b). The library was amplified in BLT5403 *E. coli* and variants were selected for their ability to bind to a lipid mixture containing ceramide 1-phosphate (C1P) [48]. The mouse PLA2-alpha cDNA was a generous gift from Michael Gelb, University of Washington. NiSepaharose Excel, capacity 10 mg/mL, was purchased from GE. Other reagents were purchased from Thermo-Fisher.

To select for C1P binding, lipid nanodiscs were developed as a bilayer affinity matrix. The His6-tagged membrane scaffold protein MSP1D1 [49] was expressed in BL21 *E. coli* from a pET28a plasmid and purified on nickel resin, then used to generate lipid nanodiscs comprising 30 mol% phosphatidylcholine, 20 mol% phosphatidylserine, 40 mol% phosphatidylethanolamine, and 10 mol% C1P [50]. To separate nanodiscs from large lipid aggregates and free protein, the mixture was subjected to gel filtration using a Superose 6 10/300 GL column (Pharmacia) and the major peak following the void volume was collected. To generate the affinity resin, 70 μg of nanodiscs (quantified by protein content) was incubated overnight at 4 °C with 10 μL nickel resin in 20 mM Tris pH 7.5 and 100 mM NaCl. The resin was washed twice in the same solution and used in phage binding reactions.

Phage expressing the C2 domain variant library were titered and diluted to a concentration of 5 × 10^9^ pfu/mL in 20 mM Tris pH 7.5 and 100 mM NaCl, then incubated with lipid nanodisc affinity resin plus 10 μM calcium in a final volume of 350 μL. After a 2-hour incubation at 4 °C, the resin was washed four times in 1 mL of the incubation buffer containing 20 mM imidazole. Phage bound to nanodiscs were eluted with 20 mM Tris pH 7.5 containing 500 mM imidazole. Phage from the elution were titered, amplified, and subjected to additional rounds of selection. Three replicate selections were performed on different days using the same input phage library.

Sequencing libraries were prepared by PCR amplifying the variable region using primers that append Illumina cluster generating and index sequences (Additional file 7) before sequencing using the Illumina NextSeq platform with a NextSeq high output kit (75 cycles, FC-404-1005). Reads were demultiplexed using bcl2fastq v2.17 (Illumina) with the arguments *bcl2fastq --with-failed-reads --create-fastq-for-index-reads --no-lane-splitting --minimum-trimmed-read-length 0 --mask-short-adapter-reads 0*. Quality was assessed using FastQC v0.11.3 [51]. Demultiplexed reads are available in the NCBI Sequence Read Archive, BioProject Accession PRJNA344387.

### Neuraminidase data analysis

Raw reads were demultiplexed using a custom script based on three-nucleotide barcodes provided by the original authors [30]. The reads were analyzed in Enrich2 v1.0.0 as ten experimental conditions: five non-overlapping 30-base regions of the neuraminidase gene in either the presence or absence of oseltamivir. Reads were required to have a minimum quality score of 23 at all positions and contain no Ns. The five mutagenized regions were scored independently and then merged to create a single set of variant scores for each treatment. To be consistent with the original study, we removed variants containing multiple nucleotide changes with the exception of p.Ile117Ter and p.Thr226Trp that were individually validated. The *p* values for comparing variant scores to wild-type in each treatment and comparing variant scores between treatments were calculated using a *z*-test. All three sets of *p* values were jointly corrected for multiple testing using the qvalue package in R [52], and variants with a *q* value of less than 0.05 were reported as significant.

### Analysis of other datasets

For previously published datasets, raw sequence files in FASTQ format were obtained from the respective authors. Datasets (Table 1) were analyzed independently using Enrich2 v1.0.0. The BRCA1 dataset was analyzed in a single run with separate experimental conditions for the yeast two-hybrid and phage display assays. For all datasets except neuraminidase, reads were required to have a minimum quality score of 20 at all positions and contain no Ns.

For the WW domain sequence function map (Fig. 1), scores and standard errors were calculated using weighted least squares linear regression in two technical replicates and the replicates were combined using the random-effects model as described.

### Enrich2 software implementation

Enrich2 is implemented in Python 2.7 and requires common dependencies for scientific Python. The graphical user interface is implemented using Tkinter. A deep mutational scanning experiment is represented as a tree of objects with four levels: experiment; condition; selection; and sequencing library. Each object’s data and metadata are stored in a single HDF5 file, including intermediate values calculated during analysis.

Enrich2 is designed to be run locally on a laptop computer and does not require a high-performance computing environment. Most analyses can be run overnight (Table 1). Run times in Table 1 were measured using a MacBook Pro Retina with 2.8 GHz Intel Core i7 processor and 16GB of RAM.

## Additional files

Additional file 1: Wild-type normalization performance table. (XLSX 9 kb)

Additional file 2: Supplementary figures. (PDF 12 kb)

Additional file 3 Replicate correlation tables. (XLSX 11 kb)

Additional file 4: Individually validated variants of the neuraminidase gene. (XLSX 9 kb)

Additional file 5: Variants with higher scores than wild-type in the presence of oseltamivir. (XLSX 11 kb)

Additional file 6: Wild-type score percentile table. (XLSX 45 kb)

Additional file 7: C2 domain primer sequences. (XLSX 8 kb)
