# Correction to: A statistical framework for analyzing deep mutational scanning data

**DOI:** 10.1186/s13059-018-1391-7

**Published:** 2018-02-07

**Authors:** Alan F. Rubin, Hannah Gelman, Nathan Lucas, Sandra M. Bajjalieh, Anthony T. Papenfuss, Terence P. Speed, Douglas M. Fowler

**Affiliations:** 1 Bioinformatics Division, The Walter and Eliza Hall Institute of Medical Research, Parkville, Australia; 2 Department of Medical Biology, University of Melbourne, Melbourne, Australia; 3 Bioinformatics and Cancer Genomics Laboratory, Peter MacCallum Cancer Centre, Melbourne, Australia; 4 Department of Genome Sciences, University of Washington, Seattle, USA; 5 Institute for Protein Design, University of Washington, Seattle, USA; 6 Department of Pathology, University of Washington, Seattle, USA; 7 Sir Peter MacCallum Department of Oncology, University of Melbourne, Melbourne, Australia; 8 Department of Mathematics and Statistics, University of Melbourne, Melbourne, Australia; 9 Department of Bioengineering, University of Washington, Seattle, USA

## Correction

After publication of our article [1] it was brought to our attention that a line of code was missing from our program to combine the within-replicate variance and between-replicate variance. This led to an overestimation of the standard errors calculated using the Enrich2 random-effects model.

This programming error has been corrected in v1.2.0 of the software, available from [2].

We have reanalyzed the data in the paper using v1.2.0. The results are very similar and do not significantly affect any of the manuscript’s conclusions. Notably, none of the numeric values quoted in the main text change.

Updated versions of Figs. 4, 5, 6 and 7 are provided below. Updated versions of Additional file 2 Figures S1, S2 and S4 and Additional files 3, 4 and 5 are available from the links below.

**Fig. 4:**
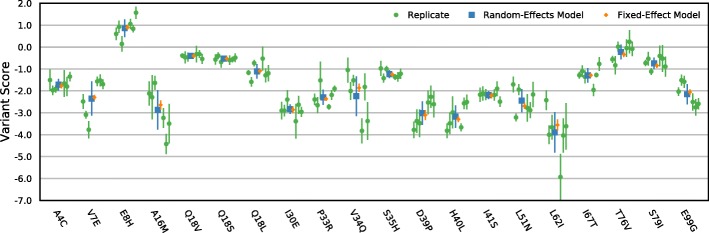
A random-effects model for scoring replicate selections. Variant scores for 20 randomly selected variants from the BRCA1 E3 ubiqutin ligase dataset are shown. The replicate scores (*green*) were determined for each variant using Enrich2 weighted regression. Combined variant score estimates were determined using a fixed-effect model (*orange*) or the Enrich2 random-effects model (*blue*). In all cases, *error bars* show +2 or −2 standard errors

**Fig. 5:**
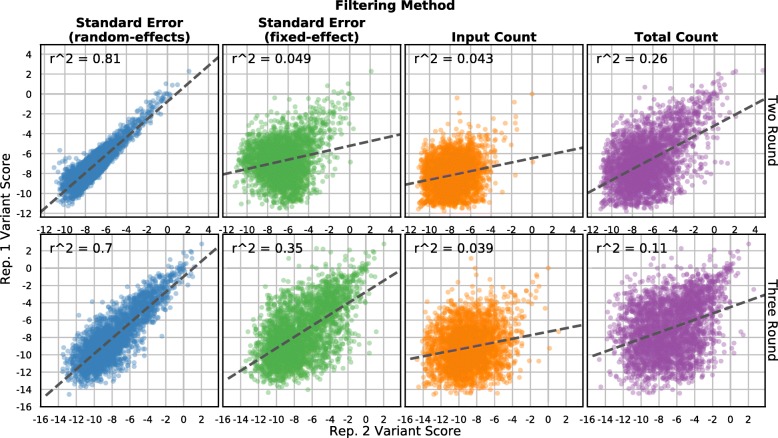
Standard error-based filtering improves replicate correlation. Variant scores from two replicates of the C2 domain dataset are shown. Each *panel* plots the top quartile of variants selected by standard error from the random-effects model (*leftmost column*, *blue points*), standard error from the fixed-effect model (*middle-left column*, *green points*), input library count (*middle-right column*, *orange points*), or total count in all libraries (*rightmost column*, *purple points*). Scores and standard errors are calculated using only the input and final round of selection (*top row*) or using all three rounds (*bottom row*). The *dashed line* is the best linear fit and the Pearson correlation coefficient is shown

**Fig. 6:**
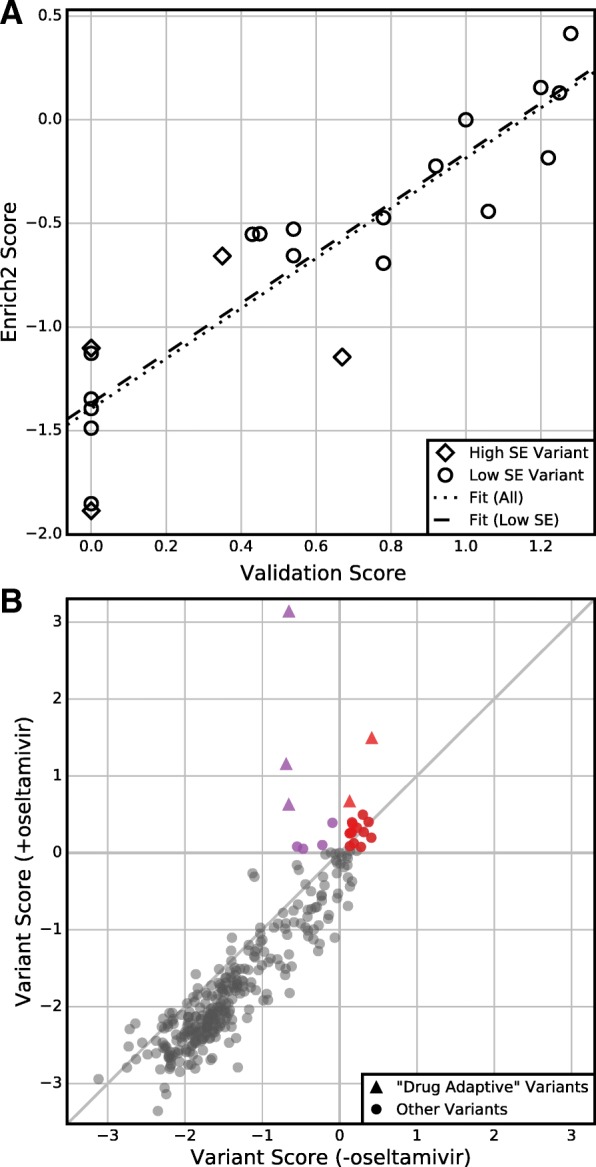
Standard errors enable hypothesis testing. **a** Enrich2 variant scores are plotted against single-variant growth assay scores for the 22 individually validated variants of the neuraminidase dataset. Four (18%) of these variants have Enrich2 standard errors larger than the median standard error. The *dotted line* shows the best linear fit for all variants and the *dashed line* shows the best linear fit for variants with standard errors less than the median. **b** Enrich2 variant scores are plotted for selections performed in the presence or absence of the small molecule inhibitor oseltamivir. *Colored points* indicate variants that significantly outperformed wild-type in the drug’s presence. *Red points* also scored significantly higher than wild-type in the drug’s absence. *Triangles* indicate the five “drug-adaptive” mutations identified originally [3]

**Fig. 7:**
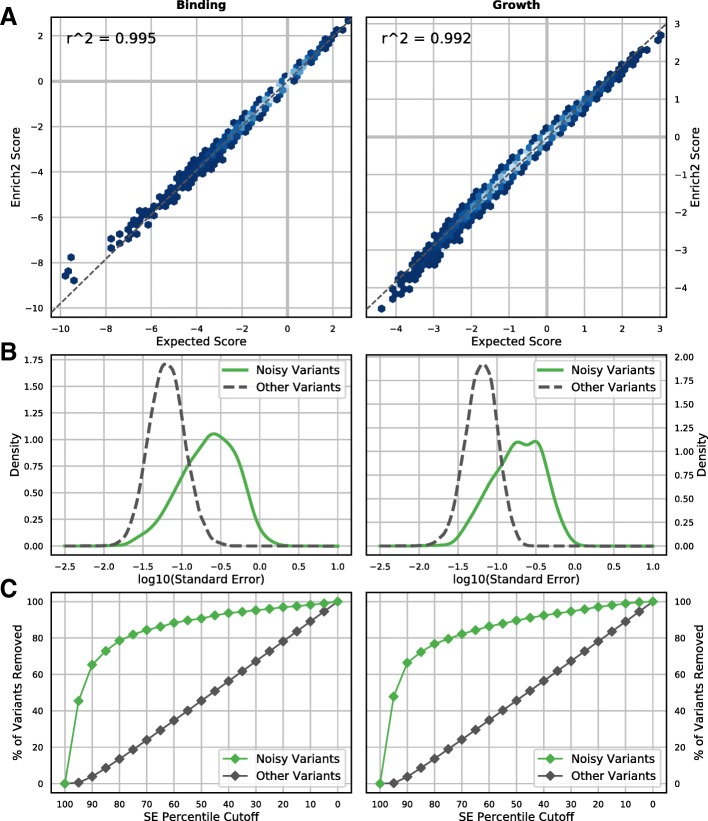
Variant scoring for growth and binding experiments using simulated data. **a** Enrich2 variant effect scores derived from simulated data are plotted against expected Enrich2 scores based on true variant effects in the simulation. Enrich2 accurately scores variants in both simulated binding assays (*left*) and growth assays (*right*). *Shading* indicates point density from low (*blue*) to high (*white*). **b** Noisy variants were generated by randomizing their true effect in one replicate selection (*green line*). Noisy variants have higher overall standard errors than other variants (*dashed gray line*) in both binding and growth assay simulations. **c** The percentage of variants removed at each standard error percentile cutoff (5% intervals) is plotted. Standard error filtering preferentially removes noisy variants (*green points*)

We would like to thank Jörn Schmiedel for alerting us to this issue, helping us reproduce it and testing the fix.

## Additional files

Additional file 2: Supplementary figures. (PDF 423 kb)

Additional file 3: Replicate correlation tables. (XLSX 14 kb)

Additional file 4: Individually validated variants of the neuraminidase gene. (XLSX 12 kb)

Additional file 5: Variants with higher scores than wild-type in the presence of oseltamivir. (XLSX 15 kb)
