# Supplementary material for: Correction to: A statistical framework for analyzing deep mutational scanning data
Source: Genome Biol. 2018 Feb 7;19:17. doi: 10.1186/s13059-018-1391-7 (PMC5803959; doi:10.1186/s13059-018-1391-7)
Supplement: Supplementary file 1 — Supplementary figures. (PDF 423 kb) [file 13059_2018_1391_MOESM1_ESM.pdf]

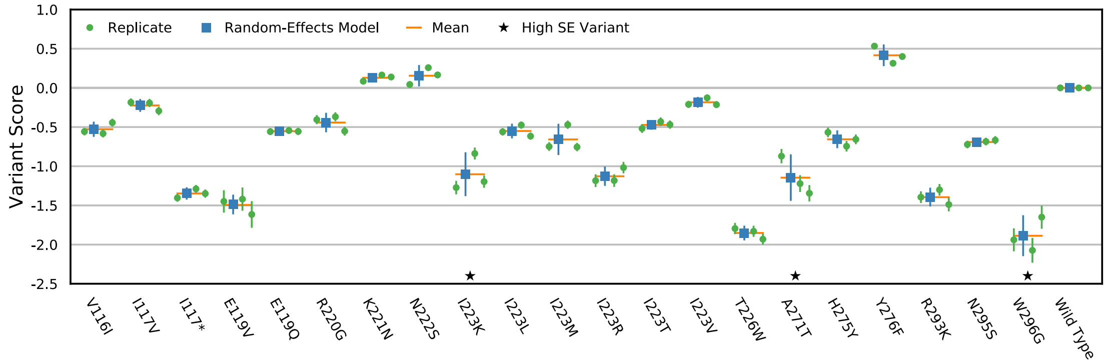

Figure S1: Random-effects model performance for individually validated neuraminidase variants. Variant scores for 22 individually validated variants from the neuraminidase dataset are shown. The variant scores for each replicate (green) are plotted along with the mean variant score (orange line) and the combined variant score from the random-effects model (blue). Error bars on the replicate and random-effects model scores show plus or minus two standard errors. Variants with high standard errors (greater than 50th percentile of scored variants with a single amino acid change) are marked with a star.

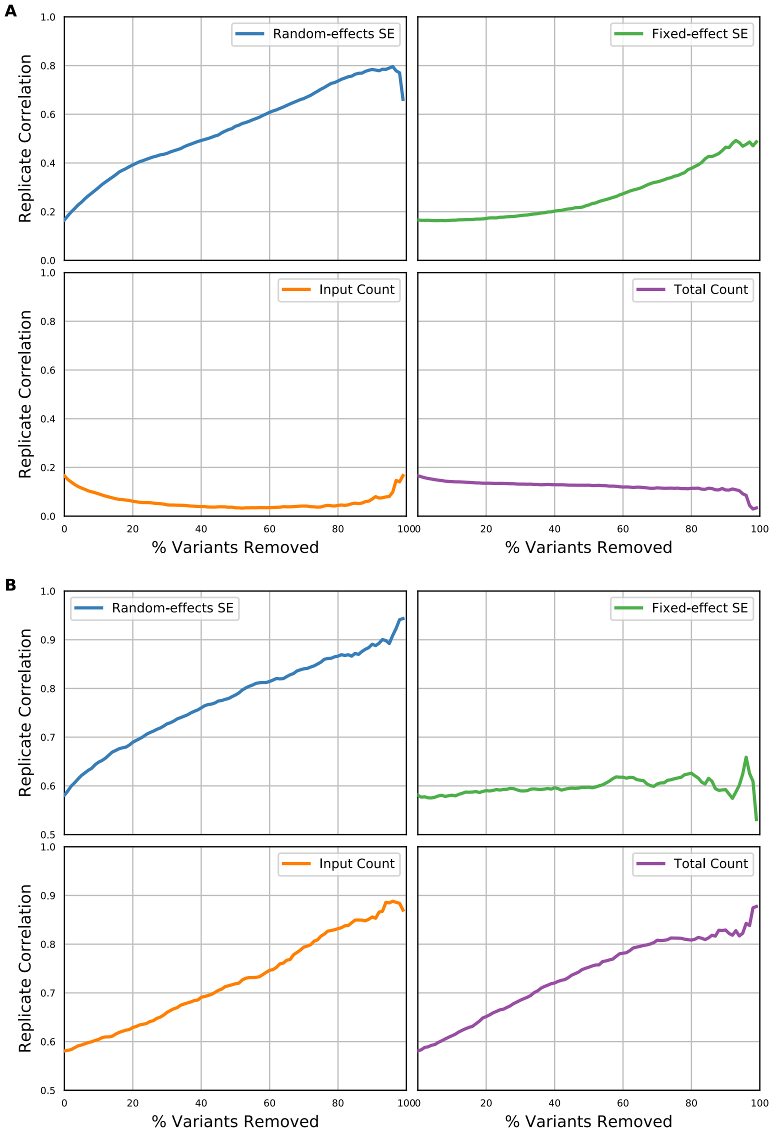

Figure S2: Removal of variants by standard error improves replicate correlation. The percentage of variants removed is plotted against the Pearson correlation coefficient (r^2) of the remaining variants in (**A**) two replicates of the C2 domain dataset and (**B**) two replicates of the BRCA1 E3 ubiquitin ligase activity dataset. Variants were removed according to one of four filtering methods: standard error from the random-effects model (**upper-left, blue**), standard error from the fixed-effect model (**upper-right, green**) input library count (**lower-left, orange**), or total count in all libraries (**lower-right, purple**). Scores and standard errors for the C2 domain data were calculated using all three rounds of selection.

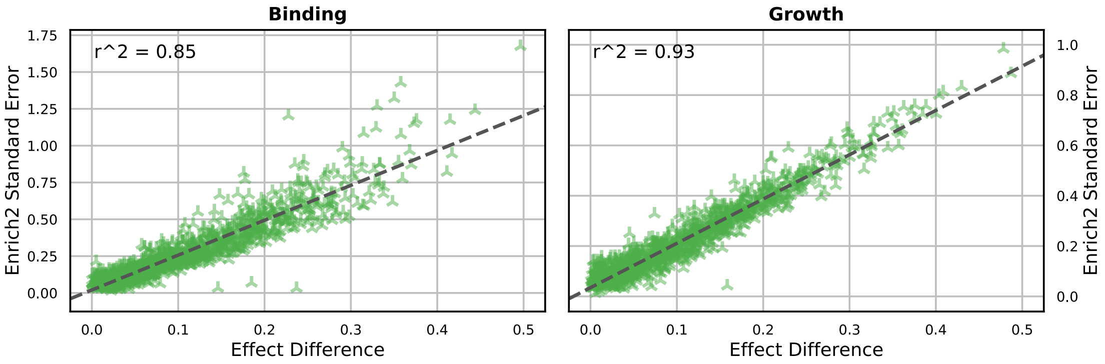

Figure S4: Effect difference in noisy variants is correlated with standard error. The standard error from the random-effects model in each of the 1,000 noisy variants is plotted against the absolute difference between the resampled true effect and original true effect. The dashed line is the best linear fit, and the Pearson correlation coefficient is shown.
